# The Impact of Internet Health Information on Patient Compliance: A Research Model and an Empirical Study

**DOI:** 10.2196/jmir.4333

**Published:** 2015-06-11

**Authors:** John Laugesen, Khaled Hassanein, Yufei Yuan

**Affiliations:** ^1^ Pilon School of Business Sheridan College Mississauga, ON Canada; ^2^ DeGroote School of Business Information Systems Area McMaster University Hamilton, ON Canada

**Keywords:** Internet health information, information asymmetry, patient compliance, patient-physician concordance, physician quality, consumer health

## Abstract

**Background:**

Patients have been increasingly seeking and using Internet health information to become more active in managing their own health in a partnership with their physicians. This trend has both positive and negative effects on the interactions between patients and their physicians. Therefore, it is important to understand the impact that the increasing use of Internet health information has on the patient-physician relationship and patients’ compliance with their treatment regimens.

**Objective:**

This study examines the impact of patients’ use of Internet health information on various elements that characterize the interactions between a patient and her/his physician through a theoretical model based on principal-agent theory and the information asymmetry perspective.

**Methods:**

A survey-based study consisting of 225 participants was used to validate a model through various statistical techniques. A full assessment of the measurement model and structural model was completed in addition to relevant post hoc analyses.

**Results:**

This research revealed that both patient-physician concordance and perceived information asymmetry have significant effects on patient compliance, with patient-physician concordance exhibiting a considerably stronger relationship. Additionally, both physician quality and Internet health information quality have significant effects on patient-physician concordance, with physician quality exhibiting a much stronger relationship. Finally, only physician quality was found to have a significant impact on perceived information asymmetry, whereas Internet health information quality had no impact on perceived information asymmetry.

**Conclusions:**

Overall, this study found that physicians can relax regarding their fears concerning patient use of Internet health information because physician quality has the greatest impact on patients and their physician coming to an agreement on their medical situation and recommended treatment regimen as well as patient’s compliance with their physician’s advice when compared to the impact that Internet health information quality has on these same variables. The findings also indicate that agreement between the patient and physician on the medical situation and treatment is much more important to compliance than the perceived information gap between the patient and physician (ie, the physician having a higher level of information in comparison to the patient). In addition, the level of agreement between a patient and their physician regarding the medical situation is more reliant on the perceived quality of their physician than on the perceived quality of Internet health information used. This research found that only the perceived quality of the physician has a significant relationship with the perceived information gap between the patient and their physician and the quality of the Internet health information has no relationship with this perceived information gap.

## Introduction

### Background

The patient-physician relationship has been noted to be second only to family relationships in terms of importance. It is viewed as extremely or very important by 67%, exceeding relationships with spiritual advisors, pharmacists, coworkers, and financial advisors [1]. The benefits of this relationship and, in turn, physician advice can only be achieved if patients follow the treatment regimens relatively closely [2]. This concept, known as compliance, is important to examine because prior studies have shown that noncompliance rates can be as high as 80% and noncompliance “creates a number of serious problems: (1) for the individual in reduced quality and quantity of life, lower income due to inability to work, and higher medical costs; (2) for society, which pays higher insurance and medical costs because noncompliant patients often require more expensive and invasive health care; and (3) for corporations because they experience lower productivity from sick and absent workers” [3]. One suggested way to improve compliance is through improved patient-physician communication [3-7], collaboration and participative decision making [2,3], and better concordance between patients and physicians with respect to medical diagnoses and treatment regimens [3,4,8-11].

Patient use of the Internet in searching for and gathering health information is growing and has now become somewhat commonplace. The Pew Internet & American Life Project reports 80% of American Internet users have searched for some type of Internet health information and millions of people search for Internet health information on a typical day [12]. However, as the use of the Internet as a source for health-related information becomes more commonplace, relations between the patient and physician can become strained [1,13-15] and this strained relationship due to Internet health information could potentially impact physician-patient concordance and patient compliance. Based on the preceding discussion, it is important to understand the true impact of patients’ increasing utilization of Internet health information on the patient-physician relationship and patients’ compliance with their treatment regimens through theoretically rigorous and empirically validated studies. However, to the best of our knowledge, no known models have been developed and empirically validated that examine patients’ compliance and concordance with their physician where Internet health information is widely available and used by patients. In fact, little research (quantitative or qualitative) on the impact of Internet health information on the patient-physician relationship and compliance has been completed. Previous studies have looked at factors that influence compliance [5,6]; however, none have looked at how patients’ perceptions of Internet health information and their physician quality impacts information asymmetry, concordance with their physician, and compliance with physician advice. This new theoretical lens is important because traditionally the patient-physician relationship was subject to the influences of information asymmetry (ie, physicians having significantly more and better health-related information), but this influence may be reduced by patient use of Internet health information.

### Compliance

The term compliance is the most common way to describe a patient following his/her physician’s treatment instructions [16]. Numerous previous research studies and reports have identified the issue of noncompliance and the importance of compliance [4,9,16] and the global problems that noncompliance are causing for health care systems [16]. Compliance is very important to study because previous research has shown that patients who are compliant exhibit better health outcomes than those who are noncompliant [17]. Noncompliance rates range from 25% to 80% [3-5,10,18] and noncompliance is estimated in the United States to cause 125,000 deaths, 19% of all hospital admissions, and more than US $100 billion in additional health care costs per year [3,6]. Noncompliance is linked to substantial worsening of disease and death [2] and is also reported to waste resources, cause preventable morbidity and mortality, and result in the loss of health care funds and productivity [19]. Given the increasing incidence of chronic illness [20], the study of compliance becomes even more important as treatment becomes more reliant on patient self-management [19]. Therefore, understanding and improving compliance can lead to better patient health outcomes [19] and lower costs of health care.

### Principal-Agent Theory and Perceived Information Asymmetry

Principal-agent theory seeks to understand and explain the association between self-interested parties who have potentially differing goals in situations where there is an imbalance of information between the parties [21]. In this theory, the principal “hires” the agent who performs some task on behalf of the principal because the principal typically has less information than the agent does (ie, information asymmetry). This theory has been applied in areas such as economics, accounting, finance, marketing, political science, organizational behavior, sociology, and buyer-seller relationships [21,22]. Previous research has applied principal-agent theory to the relationship between physicians (agents) and patients (principals) [23,24]. It is our contention that principal-agent theory applies to the patient-physician relationship (specifically in the context of Internet health information). There is a recognized asymmetry of information in the patient-physician relationship [23]. This perceived imbalance of knowledge and power has historically placed patients in a vulnerable position [25] with the flow of information between patient and physician being tenuous because of the knowledge/power gap [25]. However, the past decade (ie, the Internet health information years) has fostered a challenge to this asymmetrical model of interaction where the physician held the majority of the information and power [26]. Historically, physicians typically provided information to patients to ensure patient acceptance of the physician’s diagnoses and treatments [26]; however, this is changing given the quantity and quality of Internet health information that is available to patients.

### Internet Health Information

Patients receive medical information from physicians, but they also obtain medical information from a variety of other sources, such as friends, news, books, and now more frequently and conveniently, from the Internet. It is logical to assume that a patient’s level of knowledge/information vis-à-vis their physician is a function of the quality of their own information (which is now mainly based on information gathered from the Internet) and the quality of their physician (an element that includes physician knowledge). Therefore, this study incorporates both Internet health information quality and physician quality as key elements in both the patient’s assessment of their relative knowledge level and in the concordance between the patient and the physician. From a patient perspective, the effects of Internet health information have been shown to be both positive and negative. From a positive standpoint, the most commonly cited effect is patient empowerment, with Broom [13] indicating Internet health information can provide a sense of empowerment, purpose, and control, and patient empowerment can lead to better treatment and higher levels of patient satisfaction. Another important patient benefit from Internet health information is that it allows patient control over their rate of learning, thus reducing information overload often experienced in a physician’s office [27]. Other positive effects of Internet health information are enhanced patient confidence in dealing with physicians, better health choices and decision making, improved understanding of health conditions, and improved communication with physicians [28,29]. Improved information access through Internet health information, given the information is clinically relevant, accurate, and validated, has been linked to improved outcomes [30]. From a negative standpoint, the major issue regarding Internet health information is patient concern about physician disapproval. Patients worry that this disapproval can lead to physician hostility, irritation, and lower quality of care resulting in patient anxiety, confusion, and frustration [13].

Physicians generally accept that the Internet may lead to patients becoming better informed; however, 40% of physicians believe that this may damage the patient-physician relationship [14]. Physicians worry that the use of the Internet may lead to patient confusion, unrealistic expectations, and potential increases in litigation [14]. In addition, physicians are concerned that the patient-physician relationship can be affected when they must explain to their patients that the information they have gathered from the Internet is not accurate or complete [1] and, therefore, potentially irrelevant. Physicians are concerned about potential Internet health misinformation and, more importantly, patient misinterpretation of the Internet health information [15]. However, despite this, 90% of physicians surveyed feel that providing a greater quantity of better medical information to patients is beneficial [14]. Although physician information is the most trusted source and patients report that their preference is to go to their physician first to get information, only 10.9% of patients actually go to their physician first, whereas 48.6% go online first [31], most likely because of the accessibility, convenience, and immediacy of the information.

### Physician Quality

Although the information a patient holds (much of which is gathered through the Internet) forms one side of the information equation, the other important element a patient considers when determining their relative (to their physician’s) level of knowledge would be their perception of their physician’s competence/knowledgeability and their physician’s communication capabilities (because their perception of the physician’s knowledge can only be derived based on communications with their physician). Therefore, both physician competence/knowledgeability and communication capabilities are essential components of physician quality [32] along with physician empathy. From a health information perspective, patients report they value their physician’s knowledge more than any other health care information source, including Internet health information [27]. Therefore, it is logical to believe that physician quality plays a major role in a patient’s thought process when determining information asymmetry relative to their physician and concordance with their physician’s recommendation, which are 2 major elements of this research study.

### Research Model and Hypotheses

**Figure 1:**
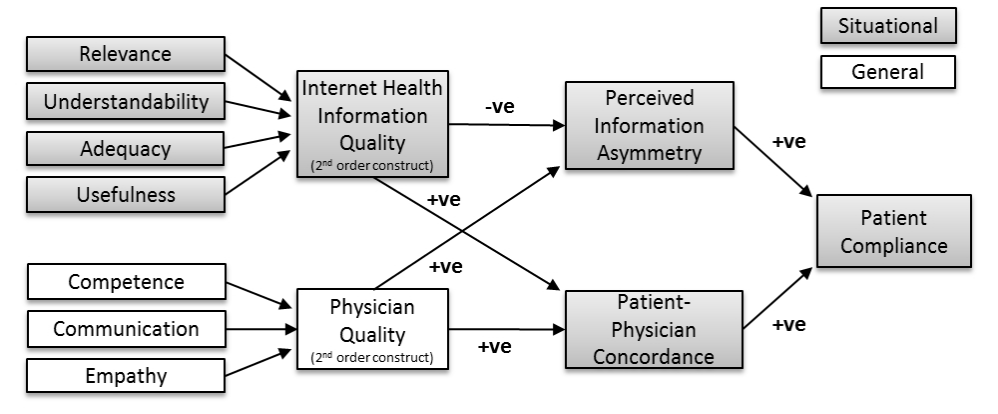
Research model.

We propose the theoretical model shown in Figure 1 to examine the impact of both patients’ use of Internet health information combined with physician quality–related factors on patients’ compliance with physician’s advice in the presence of Internet health information. Although other factors may be involved in compliance, we focus on factors related to Internet health information use and physician quality and their impact on information asymmetry and concordance as antecedents to patient compliance. The majority of the constructs in the research model are assessed on a situational basis in that the survey items used referred to a specific significant health situation. However, it was not possible for one construct (ie, physician quality) to be assessed on a single situational basis (because it would be difficult for respondents to separate their general trust in the physician from the situational trust formed regarding the significant health situation) and, therefore, physician quality was assessed on an overall basis.

### Patient Compliance

Patient compliance involves the extent to which the behavior of a patient matches the physician’s recommendations [16]. There are a number of previous studies that show compliance has relationships with a number of antecedent concepts, including the physician-patient relationship and interactions, patient-physician communication, patient knowledge and attitudes and shared decision making [3,4,6,9], concordant patient-physician relationship [4], confidence in their physician’s ability to help them and satisfaction with the concern shown by physicians [10], participative decision making [3], and physician competence [33]. Previous studies have shown that patients’ self-reports of compliance generally correspond with objective measures of compliance [16]; therefore, we used self-reported measures of compliance in this research.

### Perceived Information Asymmetry

For the purposes of this study, we have adapted the description of perceived information asymmetry put forth by Pavlou et al [21] and define perceived information asymmetry as the patients’ perception that the physician has a greater quantity and/or quality of information compared to themselves [21]. There are no known previous studies with direct theoretical support for the hypothesized relation between perceived information asymmetry and compliance. However, given that perceived information asymmetry in this research involves the information/knowledge gap between the patient and the physician, it is logical to assume that higher levels of physician information/knowledge are directly related to higher levels of information asymmetry and that patients who feel that their physician has more and/or better health-related information than they do will be more likely to comply with the physician’s recommended diagnosis and treatment regimen. Previous studies have shown a relationship between physician information/knowledge levels and compliance or, conversely, a relationship between physician knowledge deficiencies and a lack of compliance [34-36]. Solem et al [36] indicated that forming an understanding of the knowledge gaps between a physician and patient may be critical to improving patient compliance. Therefore, we hypothesized that perceived information asymmetry will have a positive impact on patient compliance.

### Patient-Physician Concordance

Patient-physician concordance involves agreement between a patient and their physician regarding the medical problem and treatment regimen [8]. In essence, concordance encompasses the agreement regarding the treatment whereas compliance involves whether or not the patient complied with the treatment, regardless of whether or not there was concordance. Prior studies support the relationship between the concept of patient-physician concordance and compliance [3,4,8-11] with Kerse et al [8] finding that patients reporting high levels of concordance were 33% more likely to be compliant in taking medications prescribed during the consultation. Another study found that better communication and concordance between a patient and their physician can lead to improved compliance [4]. A study of Korean patients found that patient-physician partnership, a concept very similar to concordance, had a very strong relationship with compliance, which was attributed to the Korean patient’s desire for an egalitarian relationship with their physician [7]. Finally, Wroth and Pathman [10] found that patient-physician concordance is associated with medication compliance. Therefore, we hypothesized that patient-physician concordance will have a positive impact on patient compliance*.*

### Internet Health Information Quality

Internet health information quality is a second-order construct comprised of perceptions of relevance (ie, clearness, relevance, and goodness), understandability (ie, clarity, understandability, and readability), adequacy (ie, sufficiency, completeness, necessity), and usefulness of the information on a health infomediary’s website [37]. Support for the relationship between Internet health information quality and information asymmetry is provided via a previous study that examined website informativeness and perceived information asymmetry and found a significant relationship between these variables. A number of studies that include information asymmetry in the accounting and financial domains suggest that better information quality is related to lower levels of information asymmetry [38,39]. Additional studies in economics suggest that the provision of better information is a potential solution to asymmetry problems (ie, to consumers reducing the level of asymmetry) [40] and that the dissemination of information (eg, through educational programs or labeling) aims at reducing issues resulting from information asymmetry [40-43]. Finally, a previous study in the context of digital information and food traceability found a significant negative relationship between informativeness (ie, the extent to which the Internet provides participants with helpful information) and information asymmetry [44]. Therefore, we hypothesized that Internet health information quality will have a negative impact on perceived information asymmetry.

Although there are no known studies that specifically examine the relationship between Internet health information quality and patient-physician concordance, it is logical to believe that patients who have accessed high-quality information regarding their medical situation will be able to have more meaningful communication with their physician, which in turn should lead to a higher level of agreement between the patient and physician regarding the medical issue and treatment. This logic is supported through studies that report that better-informed patients can lead to enhanced communication between patients and physicians [45], and that encouraging enhanced 2-way patient-physician communication may have a positive influence on concordance [46]. Finally, a study that examined the effects of providing medical information to patients found that this led to decisions that were based on both the knowledge of the physician and the patient’s preferences, which is very similar to the notion of concordance [47]. Therefore, we hypothesized that Internet health information quality will have a positive impact on patient-physician concordance.

### Physician Quality

In this research, physician quality is a second-order construct comprised of perceptions of competence, empathy, and communication [32]. This representation of physician quality encompasses both professional core physician qualities along with important personal qualities of the physician [32]. Previous studies have shown support for the relationship between the individual elements of physician quality and concordance between patients and their physicians. Janz et al [48] indicate that lower levels of concordance between patient and physician regarding treatment decisions show the need for better communication between patient and clinician. Riekert et al [49] found that poor patient-physician communication and information sharing are contributing factors of nonconcordance. A study by Vermeir et al [9] indicates that physician empathy may be an essential element of patient-physician concordance. Given that individual elements of physician quality are related to concordance, it is logical to assume that the higher the patient’s perception of their physician’s overall quality, the more likely they are to come to an agreement about the significant health situation and recommended course of action. Therefore, we hypothesized that physician quality will have a positive impact on patient-physician concordance.

Although there is no known prior research that specifically examines the relationship between physician quality and information asymmetry, the support cited previously for the hypothesized relationship between Internet health information quality and information asymmetry also plays a role in the physician quality and information asymmetry relationship. The support noted previously shows that different amounts and quality of information on each side of the agency relationship affect information asymmetry. Given that more and better patient information should reduce information asymmetry, it is logical to assume that more and better physician information would increase information asymmetry (because this construct is the gap between the patient’s and physician’s information). Therefore, we hypothesized that physician quality will have a positive impact on perceived information asymmetry.

## Methods

### Instrument Development

This research made use of previously validated instruments to measure the constructs in the model, as per Boudreau et al [50]. Unless otherwise noted (see Multimedia Appendix 1), all items were measured using a 7-point Likert scale with ranges from strongly agree to strongly disagree. Compliance was measured using a 5-item scale adapted from Hausman [6]. Respondents were allowed to indicate “not applicable” to individual compliance questions because not all patients would be required to follow each and every one of the directions noted in the survey items (eg, some patients would not be required to take medications as part of the treatment regimen; therefore, these participants would need the ability to indicate not applicable to this question). Patient-physician concordance was measured using a 5-item scale adapted from Kerse et al [8], which was designed to assess agreement between physician and patient. Perceived information asymmetry was measured using a 4-item scale developed based on items from Pavlou et al [21] and Dunk [51]. These items were adapted to specifically address the context of the information gap between the physician and the patient regarding the significant health situation.

Physician quality was developed as a second-order construct comprised of competence, empathy, and communication. These elements of physician quality are based on Jayanti and Whipple [32] that describe physician quality as a function of listening skills (ie, communication), competence/knowledgeability, and empathy. For this second-order construct, there were no known scales that specifically addressed competence. Therefore, we adapted the validated McKnight et al [52] competence scale (which addressed competence in the legal profession) to a physician competence context. Given both contexts (ie, legal and medical) are professional ones, the McKnight et al [52] scale was deemed to be the most applicable for this research. The empathy items were adapted from Kim et al [7], a study that included an examination of the relationship between physician empathy and patient compliance. The communication items were adapted from Hausman [6], a study that examined physician communication and its relationships with both patient participation in the decision-making process (similar to concordance) and patient compliance with physician advice.

Finally, Internet health information quality was developed as a second-order construct comprised of adequacy, understandability, usefulness, and relevance. These 4 areas were each measured using 4-item scales adapted from Zahedi and Song [37]. The validated scales contained in Zahedi and Song [37] were highly applicable to this study because they specifically measured trust and quality in an online health information provider context. The final set of survey items is included in Multimedia Appendix 1.

### Second-Order Constructs

Second-order constructs are used in this research to model (1) Internet health information quality because this variable is comprised of the first-order quality factors of usefulness, adequacy, relevance, and understandability [37] and (2) physician quality because this variable is comprised of competence, empathy, and communication [32]. A full statistical analysis of the second-order constructs is provided in Multimedia Appendix 2. As per Chin [53]: “Higher order latent variables are often useful if a researcher wishes to model a level of abstraction higher than those first-order constructs used in a basic [covariance-based structural equation modeling] CBSEM and [partial least squares] PLS model.” Both Internet health information quality and physician quality are structured as second-order factor models, with the direction of the relationship flowing from the first-order constructs to the second-order construct (see Multimedia Appendix 2). This model structure is characterized as reflective first-order, formative second-order as per Jarvis [54], which is the most common structure in Information Systems literature [55]. Careful consideration was given when determining to model both Internet health information quality and physician quality as second-order constructs, specifically in that the first-order factors were conceptually related to the other factors in the model and that the second-order factor fully mediated the relationships of the first-order factors in the theoretical model [53]. A number of previous Information Systems studies have made use of second-order constructs [56-59].

From a statistical perspective, this research used the indicator reuse technique proposed by Wold [60] as described in Ringle et al [55]. Specifically, “When using the PLS-SEM method for model estimation, all latent variables—which includes higher order components—must have a measurement model with at least one indicator...This approach works best when all lower order components have the same number of indicators. Otherwise, the interpretation of the relationships between the lower and the higher order components must account for the bias of unequal numbers of indicators in the lower order components” [55]. This research ensured these requirements were met with each latent variable in the model having at least one indicator and all lower order components (ie, first-order constructs) containing the same number of indicators.

### Analysis Tool Selection

This research used the second-generation statistical technique of structural equation modeling (SEM), specifically PLS implemented via Smart-PLS software version 2.0.M3. As described by Gefen et al [61], “the intricate causal networks enabled by SEM characterize real-world processes better than simple correlation-based models. Therefore, SEM is more suited for the mathematical modeling of complex processes to serve both theory...and practice.” All preanalyses with respect to data screening (ie, missing data, outliers, and multivariate statistical assumptions) were completed based on well-known statistical methods [62-65]. Once the data screening process was complete, an SEM analysis comprised of both examination and assessment of the measurement model (see Multimedia Appendix 2) and structural model, as well as additional analyses (ie, common method bias, post hoc) was completed. Overall, the SEM analysis followed the guidelines set forth by SEM and PLS experts [53,66-69].

## Results

### Recruitment

Given that this study primarily focused on the effects that Internet health information has on medical compliance, survey participants were required to have (1) recently seen a physician regarding a recent significant health situation that they were able to clearly recall their interactions with the physician for and (2) a clear recall of their experience in a search they carried out for Internet health information regarding the significant health situation in question. The qualifying questions’ use of the phrase “significant health situation” was kept general (ie, no definition or examples of significant health situation were provided) because the interpretation of significant health situation is different for different people. The most important element of this aspect of the research is that the participant deemed the health situation to be significant. Data for this research study were collected in January 2013. Given the specific characteristics required for participants in this study, a decision was made to recruit research participants via the use of a well-known research firm (ie, Research Now). Participants were randomly selected from a pool of potential respondents contained in the database of this research firm. Ethics approval for research involving human subjects was obtained from the McMaster University Research Ethics Board (Hamilton, ON, Canada) and informed consent for all participants was obtained after the nature and possible consequences of the study were explained. All ethics requirements were enforced by Research Now and participants were compensated based on Research Now policies and procedures. A total of 234 participants were recruited.

Potential participants for this study were randomly selected from the Canadian adult population. A set of prequalifying questions was used to ensure that selected participants were able to recall a search for and use of Internet health information in the recent past for a significant health situation. In addition, selected participants were required to have recently seen a physician for this significant health situation and to be able to recall their interactions with their physician regarding the significant health situation. These prequalifying criteria were very important because this study examined how the use of Internet health information impacted elements of interactions between patients and their physicians regarding a significant health situation. A total of 1418 potential participants were contacted with 234 of these meeting the prequalifying criteria. The demographics for the research sample are shown in Table 1. The demographics of the participants in this study closely matched the demographics of the population that searched and used Internet health information (ie, higher proportion of females, younger, higher education levels, higher incomes [12,70]), thus providing confirmation that we had a representative and relevant sample for this study.

Based on an outlier analysis that examined both univariate and multivariate outliers, a total of 9 cases were removed from the dataset leaving 225 usable surveys retained for further analysis. The 9 cases removed represented less than 4% of the total cases, which can be considered an acceptable amount removed from the dataset [64]. There were no missing values for the constructs in the model and a limited number of missing values identified among the control variables. The mean imputation method was used to handle the missing control variable values as per Hair et al [63] and Meyers et al [64]. Finally, a complete multivariate statistical assumptions analysis (ie, linearity, normality, and homoscedasticity) revealed no substantive issues; therefore, the dataset was deemed viable for further statistical analysis.

**Table 1:** Sample demographics (N=225).

Demographic characteristic		n (%)
**Gender**		
	Male	83 (38.4)
	Female	133 (61.6)
**Age**		
	<20	0 (0.0)
	20-29	28 (12.6)
	30-39	37 (16.6)
	40-49	47 (21.1)
	50-59	48 (21.5)
	60-69	45 (20.2)
	70-79	17 (7.6)
	≥80	1 (0.4)
**Education (highest level)**		
	High school	9 (4.2)
	Some college/university or college/university degree	139 (64.3)
	Graduate degree	68 (31.5)
**Income (Can $)**		
	<$10,000	5 (2.6)
	$10,000-$24,999	12 (6.2)
	$25,000-$49,999	37 (19.2)
	$50,000-$74,999	55 (28.5)
	>$75,000	84 (43.5)

### Statistical Analysis

**Figure 2:**
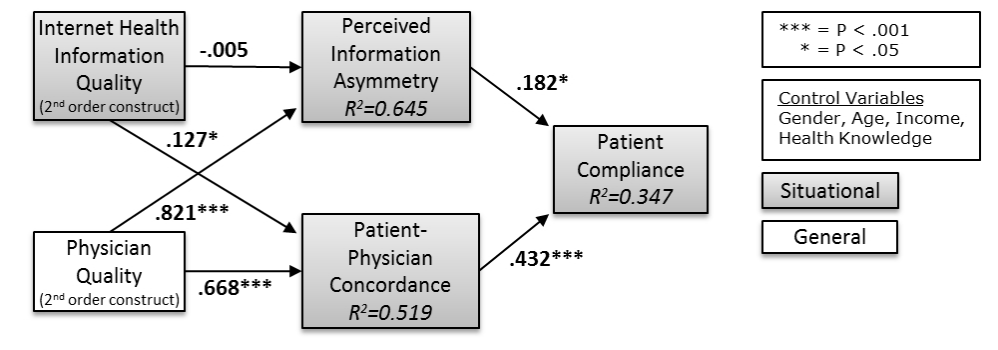
Partial least squares structural model results.

A complete control variable analysis was completed prior to the analysis of the research model. This analysis showed that 4 of the control variables (ie, age, gender, income, and health knowledge) had significant relationships with 1 or more of the endogenous constructs in the model; therefore, these control variables were included in the final structural model to ensure that the effects of these extraneous variables were accounted for. The results of the structural model are shown in Figure 2. Given the focus of PLS analysis is on prediction, an examination of the variance of the dependent measures through the *R* ^*2*^ results was completed. The results of this analysis showed moderate to substantial predictive powers based on the 0.19 (minimum), 0.33 (moderate) and 0.67 (substantial) thresholds [71], as shown in Table 2. In addition, an examination of the effects of the control variables was completed, indicating that the control variables had limited effects on the research model results as shown in Table 2.

**Table 2:** Multivariate coefficient of determination (*R* ^*2*^) results.

Endogenous construct	*R* ^*2*^		Control variable effects		
	With control variables	Without control variables	∆*R* ^*2*^	ƒ^2^	Effects
Compliance	0.347	0.333	0.014	0.021	Small
Patient-physician concordance	0.519	0.486	0.033	0.069	Small
Perceived information asymmetry	0.645	0.577	0.068	0.192	Medium

An assessment of the path estimates in the model via the magnitude and significance of the path coefficients is shown in Table 3. This assessment revealed that 5 of 6 hypotheses were fully supported with 3 of these significant at the *P*<.001 level. The *t* tests for significance were produced through the bootstrap method with the number of cases equal to the number of observations in the sample (ie, 225) and the number of samples set to 5000. For all supported hypotheses, the hypothesized algebraic sign was consistent with the path coefficient results. The lone hypotheses that was not supported (ie, Internet health information quality will have a negative impact on perceived information asymmetry) showed that the path coefficient between Internet health information quality and perceived information asymmetry was not significant and was in fact very close to zero. This perplexing result is discussed further in the Discussion section. In addition to the direct paths in the model, the 2 indirect paths (ie, Internet health information quality compliance and physician quality compliance) were also found to be significant as shown in Table 3.

**Table 3:** Partial least squares path analysis for direct and indirect effects.

Hypothesis		Path coefficient	*t*_224_	*P*
**Direct effects**				
	Perceived information asymmetry positively affects compliance	0.182	2.281	.02
	Patient-physician concordance positively affects compliance	0.432	5.868	<.001
	Internet health information quality negatively affects perceived information asymmetry	–0.005	0.121	.90
	Internet health information quality positively affects patient-physician concordance	0.127	2.371	.02
	Physician quality positively affects perceived information asymmetry	0.821	23.476	<.001
	Physician quality positively affects patient-physician concordance	0.668	13.946	<.001
**Indirect effects**				
	Internet health information quality affects compliance	0.054	2.139	.03
	Physician quality affects compliance	0.438	6.773	<.001

Effect sizes as per Cohen’s ƒ^2^ [72] were calculated and are provided in Table 4. Effect sizes aid in evaluating the impact that the antecedent constructs have on the dependent constructs and can be assessed as small (ie, 0.02), medium (ie, 0.15), or large (ie, 0.35) effect sizes based on guidelines from Roldán and Sánchez-Franco [69]. The effect size analysis clearly shows the strong impact that physician quality had in the research model with large effect sizes on patient-physician concordance and perceived information asymmetry, whereas the effect sizes of Internet health information quality were either small or not significant. In addition, the impact of patient-physician concordance on compliance was strong with a medium effect size noted, whereas the impact of perceived information asymmetry on compliance was small.

A goodness-of-fit analysis to examine model performance for both the measurement and the structural model was calculated as per Tenenhaus et al [73]. This analysis produced a single value that can be interpreted similarly to the interpretation of effect sizes as per Wetzels et al [59]. The goodness-of-fit index for this study was 0.616, which can be considered a large effect and supports the conclusion that this model performed well.

**Table 4:** Partial least squares effect size analysis.

Dependent and independent constructs		*R* ^*2*^		∆*R* ^*2*^	ƒ^2^	Effect size
		In	Out			
**Compliance**						
	Patient-physician concordance	0.347	0.237	0.110	0.168	Medium
	Perceived information asymmetry	0.347	0.327	0.020	0.020	Small
**Patient-physician concordance**						
	Internet health information quality	0.519	0.504	0.015	0.031	Small
	Physician quality	0.519	0.115	0.404	0.404	Large
**Perceived information asymmetry**						
	Internet health information quality	0.645	0.645	0.000	0.000	NS
	Physician quality	0.645	0.035	0.610	0.610	Large

### Post Hoc Analysis

All additional demographic significant health situation, health status, and health knowledge variables that were captured in the survey were examined to determine if they had significant relationships with constructs in the research model. This analysis revealed that age had a significant positive relationship with both compliance and patient-physician concordance in that the older a person was, the more they tended to come to an agreement with the physician on the medical problem or need and its management and the more likely they were to comply with the physician’s instructions. Income had a significant negative relationship with patient-physician concordance in that the higher a person’s income was, the less likely they were to believe that there was agreement between themselves and the physician regarding the significant health situation. Gender had a significant relationship with perceived information asymmetry in that females were more likely to see a smaller gap in knowledge between themselves and the physician (regarding the significant health situation) than males. Finally, overall health knowledge had a significant negative relationship with perceived information asymmetry with those individuals who identified themselves as having higher overall knowledge about their health more likely to see a lower level of information asymmetry (ie, a smaller gap in knowledge between themselves and the physician with regards to the significant health situation).

## Discussion

### Principal Results

This research has several important theoretical contributions in the field of physician-patient relationship management and important implications for practitioners (ie, both physicians and Internet health information providers). First, we found that physician quality has the most significant impact directly on patient-physician concordance and information asymmetry as well as indirectly on compliance. This finding is similar to the results of Zolnierek and DiMatteo [5], who found that enhanced physician qualities can lead to better compliance. The implication of our finding is very important for physicians in that improved compliance can be achieved through physician quality attributes of competence, communication, and empathy. This finding is supported by Kim et al [7] who found a relationship between physician expertise and compliance, and that a patient’s assessment of physician empathy significantly influenced patient satisfaction and compliance [7]. Once the physician has established their knowledge and has the ability to effectively communicate this knowledge to the patient, patients will understand the magnitude of the physician’s knowledge and be more willing to come to an agreement regarding aspects of the patient’s medical situation (eg, diagnosis, treatment options) Once this physician quality is established, enhanced patient compliance is more likely to occur because the patient will be more likely to follow the physician’s advice.

Second, the findings regarding Internet health information quality were surprising. Although Internet health information quality has a significant positive relationship with concordance between patients and physicians, the strength of this relationship is somewhat low. This indicates that although Internet health information quality has some effect on the concordance process, physician quality has a much stronger relationship and larger effect size. The implications of this finding are important for physicians, who should focus on their personal and professional skills to improve the concordance process rather than overly concern themselves with Internet health information. Given there is a significant relationship between Internet health information quality and concordance, physicians should also encourage patients to make use of Internet health information to allow for a more concordant patient relationship. A number of previous studies support this finding, suggesting that physicians encourage Internet health information usage and share the responsibility for gathering knowledge regarding their health [15], perhaps even directing patients to reputable and relevant Internet health information websites [1]. In addition, physicians are encouraged to improve their communication skills to facilitate discussions about the Internet health information brought to them by patients [29]. The significant indirect relationship between Internet health information quality and compliance is interesting in that better Internet health information quality can lead to enhanced compliance. This finding was supported by Iverson et al [27] who also found this relationship. These results suggest that both Internet health information providers and physicians can play a role in helping to improve compliance. Internet health information developers should ensure their information is adequate, understandable, useful, and relevant to potential readers, whereas physicians can encourage patients to visit high-quality, reputable, and relevant Internet health information websites to improve compliance. It is interesting that patient-gathered high-quality Internet health information is positively related to their compliance with the physician’s instructions, suggesting that better quality Internet health information most likely is in agreement with the physician’s knowledge and directives.

The nonsignificant relationship between Internet health information quality and information asymmetry was very surprising and warrants a more detailed discussion. As noted previously, information asymmetry in this study is defined as the differential between the patient’s perceptions regarding their own knowledge and their perceptions regarding their physician’s knowledge, specifically regarding the patient’s current significant health situation. Therefore, one would expect that higher levels of physician quality (including competence/knowledgeability) would increase perceived information asymmetry (a hypothesis that was supported) and that better quality Internet health information accessed by the patient should decrease the perceived level of information asymmetry between the patient and physician from the patient’s perspective. However, this was not the result found in our study. Two reasons are suggested for this perplexing finding, as discussed subsequently.

Patients will most likely increase their medical knowledge from general access Internet health information websites. However, this information is often limited to the basic understanding of medical terminologies, diagnoses, and treatments. More detailed information (eg, from academic medical journals, research papers) is typically not available to the general population, especially for more rare and/or serious conditions. Therefore, although the patient may feel they have dramatically increased their level of knowledge by reading Internet health information, they will realize during their interaction with the physician that they have simply accessed basic information that their physician is already aware of and, therefore, there would be no change in information asymmetry. Although high-quality Internet health information will most likely dramatically alter the patient’s perception of their knowledge level, once the patient discovers that the physician was already aware of this information and can fully explain why the information applies or potentially does not apply to the patient’s condition, the patient will realize that there is still a large differential between their knowledge and the physician’s knowledge. As anecdotal evidence of this phenomenon, one of the authors of this study experienced this exact situation, whereby the researcher accessed and discussed relevant high-quality Internet health information with his physician. The physician was already aware of this Internet health information and was able to clearly explain why it did not apply and how other more relevant research and medical information applied to the situation. Thus, the author left the physician’s office with the perception that the gap in knowledge between himself and the physician was quite substantial even after accessing what he thought was relevant high-quality Internet health information.

Patients who access high-quality Internet health information are most likely accessing medical information on the Internet that has been developed by physicians. For example, well-known Internet health information sites such as WebMD and HealthCentral contain information either written by physicians or based on information that writers gather from physicians. Therefore, much of the information gleaned from these general access websites would be consistent with the information that the patient’s physician already has and, thus, there would be no change in information asymmetry. More complex information from medical academic journal websites that the typical physician may not be aware of yet is not typically available to the general public and would most likely not have been a factor in the Internet health information research completed by patients.

Third, we found information asymmetry has some impact on compliance, but its impact is much weaker than the impact concordance has on compliance. This finding suggests that it is not the sheer volume of knowledge or the differential in physician versus patient knowledge that is most important in ensuring compliance, but rather the participative and concordant interactions between the patient and physician that will lead to compliance. This finding is important for both physicians and developers of Internet health information. For physicians, taking steps to ensure patient interactions are concordant and not confrontational can lead to compliance. This means that empathetic communication with patients while demonstrating competence is one of the keys to compliance. In addition, listening to the patient and the potential Internet health information–based knowledge they bring with them to the appointment can also enhance compliance. The findings also suggest that physicians need to be ready to have an open and honest discussion regarding the patient-researched Internet health information and not simply discount the potential knowledge and information that the patient brings to the discussion. Although concordance has a large effect on compliance, the weaker yet significant relationship between information asymmetry and compliance needs to be understood and addressed by physicians. A certain degree of information asymmetry between the patient and physician needs to be maintained to ensure the physician’s advice is respected and to persuade the patient to accept the physician’s professional advice. If a physician is not viewed as an expert regarding the health situation, they may lose their professional advantage and cause patients to underestimate their need for his/her medical services.

### Limitations and Future Research

As with most research, this research also has some limitations. First, this research used a cross-sectional survey that collected data from respondents at one point in time and, therefore, may not capture the full magnitude of the Internet health information use or actual compliance. In addition, cross-sectional studies do not allow definitive conclusions regarding causal inferences. We did not conduct a longitudinal research study to actually monitor the changes in patient medical knowledge, the impact of such change, and actual compliance. A longitudinal investigation may help to gain a more comprehensive understanding of both information asymmetry and compliance. It is recommended that future research include a longitudinal study to follow up with respondents on both their levels of knowledge and actual compliance. Secondly, this research did not capture partial compliance, where patients may follow physician advice but not the full course of that advice (eg, patient is prescribed medication for 10 days but stops after 8 days). The use of a 7-point Likert scale for compliance responses will have controlled much of this phenomenon because it allowed respondents to indicate if they fully, partially, or did not comply. In addition, this research included patient self-reports of compliance rather than monitoring actual compliance. Although monitoring actual compliance (and ensuring this compliance was complete and not partial) was not accomplished, previous research supports the use of compliance self-reporting and found that patient self-reports of compliance corresponded with actual compliance [16]; therefore, it is not believed that this limitation affected the results of this study. However, future research may wish to use actual monitored compliance (eg, through confirmation of follow-up appointments/treatments/tests, objective measures of taking medication) to eliminate any potential effects this may have on the results. Third, this research used patient self-reports of the significant health situation and Internet health information search recall and, therefore, there are no guarantees that patients were able to fully and accurately recall these events. However, all efforts were made to ensure only participants who were able to clearly recall the significant health situation, physician interactions, and the Internet health information search were included in this research study. In fact, a large number (ie, 84%) of potential participants were excluded from this research due to their inability to recall the required events (ie, significant health situation, physician interaction, and Internet health information search). Fourth, this research relied on patient’s assessment of the quality of the Internet health information they accessed and not the actual quality. Future research may wish to present participants with verified quality Internet health information to ensure that all participants are reporting information asymmetry, concordance, and compliance after accessing validated Internet health information. This research would allow us to isolate the effects that quality validated Internet health information has in this research model; however, this would not reflect reality because patients typically search health-related websites of varying quality. Finally, this research endeavored to match participant demographics (eg, gender, age, education, income) to the demographics of the population that search and use Internet health information. This was done to ensure that the results of this research would be generalizable to the population of people who typically use Internet health information. However, this may reduce the generalizability to the population at large. Future research may wish to recreate this study with a sample that matches the demographics of the overall population and not simply the current characteristics of typical Internet health information users.

### Conclusions

Overall, it should be strongly emphasized that our findings suggest that physician quality was the most important element in our research model, with highly significant relationships and medium to large effect sizes on information asymmetry, concordance, and ultimately compliance. This suggests that physician quality dominates the impact on these factors and physicians are encouraged to spend less time distressing about the negative impacts of Internet health information and more time improving their competence, communication, and empathy characteristics. This being said, patients should also be encouraged, both by their physicians and society (perhaps via government initiatives), to seek out and make use of high-quality Internet health information in their discussions with medical professionals. By combining both of these recommendations, improved compliance and its related benefits are more likely to occur.

## Appendix

Multimedia Appendix 1 Measurement instruments.

Multimedia Appendix 2 Statistical analysis details.

## Abbreviations

PLS: partial least squares
SEM: structural equation modeling
