# Supplementary material for: The Impact of Internet Health Information on Patient Compliance: A Research Model and an Empirical Study
Source: J Med Internet Res. 2015 Jun 11;17(6):e143. doi: 10.2196/jmir.4333 (PMC4526934; doi:10.2196/jmir.4333)
Supplement: Multimedia Appendix 1 [file jmir_v17i6e143_app1.pdf]

Measurement Instruments

Construct	Scale/Scoring	Items
Compliance (COMPLY)[i]	• 7-point Likert<br>• Strongly Agree to Strongly Disagree	1. I am following/did follow the doctor's suggestions exactly.<br>2. I am following/did follow the doctor's drug/medication recommendations.<br>3. I am following/did follow the doctor's orders, such as to stay in bed.<br>4. I have returned or plan to return to the doctor on the schedule he/she suggested.<br>5. I have had or plan to have the follow-up tests recommended by the doctor.
Perceived Information Asymmetry (PIA) [ii, iii]	• 7-point Likert<br>• Strongly Agree to Strongly Disagree	1. In comparison to me, this doctor is/was in possession of more information regarding the specific health situation<br>2. In comparison to me, this doctor is/was more familiar with the specific health situation.<br>3. In comparison to me, this doctor is/was able to better assess the specific health situation.<br>4. In comparison to me, this doctor has/had a better understanding of the specific health situation.
Patient-Physician Concordance (PPC)[iv]	• 7-point Likert<br>• Not At All to Completely	1. To what extent do you think the doctor understood why you came in to see him/her?<br>2. To what extent did you and the doctor agree on the diagnosis with respect to the significant health situation?<br>3. To what extent did you and the doctor agree on what part you play in making decisions about the significant health situation?<br>4. To what extent did you and the doctor agree on the recommended treatment for the significant health situation?<br>5. To what extent did you and the doctor agree on the possible outcomes associated with the recommended treatment for the significant health situation?
IHI Quality (IQI-QUAL)[v]	• 7-point Likert<br>• Varied (Not At All to Very and Did Not At All Contain to Completely Contained)	**Relevance:**<br>1. For your health information needs, to what degree do you believe the Internet Health Information (IHI) provided by the website was applicable to your needs.<br>2. For your health information needs, to what degree do you believe the Internet Health Information (IHI) provided by the website was related to your needs.<br>3. For your health information needs, to what degree do you believe the Internet Health Information (IHI) provided by the website was pertinent to your needs.<br>4. For your health information needs, to what degree do you believe the Internet Health Information (IHI) provided by the website was relevant to your needs.<br>**Understandability:**<br>1. For your health information needs, to what degree do you believe the Internet Health Information (IHI) provided by the website was clear in meaning.<br>2. For your health information needs, to what degree do you believe the Internet Health Information (IHI) provided by the website was easy to read.

Construct	Scale/Scoring	Items
		3. For your health information needs, to what degree do you believe the Internet Health Information (IHI) provided by the website was easy to comprehend.<br>4. For your health information needs, to what degree do you believe the Internet Health Information (IHI) provided by the website was understandable.<br>**Adequacy:**<br>1. For your health information needs, to what degree do you believe the Internet Health Information (IHI) provided by the website was sufficient.<br>2. For your health information needs, to what degree do you believe the Internet Health Information (IHI) provided by the website was complete.<br>3. For your health information needs, to what degree do you believe the Internet Health Information (IHI) provided by the website was adequate.<br>4. For your health information needs, to what degree do you believe the Internet Health Information (IHI) provided by the website contained the necessary topics/categories.<br>**Usefulness:**<br>1. For your health information needs, to what degree do you believe the Internet Health Information (IHI) provided by the website was informative.<br>2. For your health information needs, to what degree do you believe the Internet Health Information (IHI) provided by the website was valuable.<br>3. For your health information needs, to what degree do you believe the Internet Health Information (IHI) provided by the website was helpful.<br>4. For your health information needs, to what degree do you believe the Internet Health Information (IHI) provided by the website was useful.
Physician Quality (P-QUAL)[vi, vii, viii]	• 7-point Likert<br>• Strongly Agree to Strongly Disagree	**Competence:**<br>1. This doctor is competent and effective in providing medical/health advice and treatment.<br>2. This doctor performs his/her role of providing medical/health advice and treatment very well.<br>3. This doctor is a capable and proficient medical/health advice and treatment provider.<br>4. This doctor is very knowledgeable about medical and health related information.<br>**Empathy:**<br>1. This doctor respects my feelings.<br>2. This doctor shows interest in my issues.<br>3. This doctor shows concern for my wellbeing.<br>4. This doctor cares about me.<br>**Communication:**<br>1. Communication between me and this doctor is excellent.<br>2. This doctor is willing to share all relevant information with me.<br>3. There is good communication between this doctor and myself.<br>4. This doctor is willing to answer all my questions.

[i] *Hausman A. Modeling the Patient-Physician Service Encounter: Improving Patient Outcomes. Journal of the Academy of Marketing Science 2004 Oct 01; 32(4):403-417*

[ii] *Pavlou P, Liang H, Xue Y. Understanding and mitigating uncertainty in online exchange relationships: A principal-agent perspective. MIS Quarterly 2007 Mar; 31(1):105-136*

[iii] *Dunk AS. The effect of budget emphasis and information asymmetry on the relation between budgetary participation and slack. Accounting Review 1993 Apr; 68(2):400-410*

[iv] *Kerse Ngaire, Buetow Stephen, Mainous Arch G, Young Gregory, Coster Gregor, Arroll Bruce. Physician-patient relationship and medication compliance: a primary care investigation. Ann Fam Med 2004; 2(5):455-61*

[v] *Zahedi F, Song J. Dynamics of Trust Revision: Using Health Infomediaries. Journal of Management Information Systems 2008 May 12; 24(4):225-248*

[vi] *McKnight DH, Choudhury V, Kacmar C. Developing and Validating Trust Measures for e-Commerce: An Integrative Typology. Information Systems Research 2002 Sept; 13(3):334-359*

[vii] *Kim Sung Soo, Kaplowitz Stan, Johnston Mark V. The effects of physician empathy on patient satisfaction and compliance. Eval Health Prof 2004 Sept; 27(3):237-51*

[viii] *Hausman A. Modeling the Patient-Physician Service Encounter: Improving Patient Outcomes. Journal of the Academy of Marketing Science 2004 Oct 01; 32(4):403-417*
