# Supplementary material for: The Impact of Internet Health Information on Patient Compliance: A Research Model and an Empirical Study
Source: J Med Internet Res. 2015 Jun 11;17(6):e143. doi: 10.2196/jmir.4333 (PMC4526934; doi:10.2196/jmir.4333)
Supplement: Multimedia Appendix 2 [file jmir_v17i6e143_app2.pdf]

Statistical Analysis Details

Statistical Analysis – Second-Order Constructs

Given the use of second order constructs in this research model, an assessment of the proposed second order models for Internet Health Information Quality and Physician Quality were made following the methodology used by Pavlou and Gefen [i]. Specifically, the coefficients for each first order factor were modeled in a formative (i.e., not reflective) relationship with the latent second order factors using Principal Components Factor Analysis [ii]. The results of this assessment (see Figure A2-3) indicate that all first order factors are highly significant (i.e., $p < .001$), thus supporting the use of second order Internet Health Information and Physician Quality constructs in this research model. In addition, a complete validity and reliability analysis performed on the first order constructs indicated no loading or cross loading issues, strong Average Variance Extracted values (i.e., all greater than .839), Composite Reliabilities (i.e., all greater than .954) and Cronbachs α scores (i.e., all greater than .935). In summary, a thorough statistical analysis supports the use of the second order constructs of Internet Health Information Quality and Physician Quality to fully represent the multiple facets of these variables, and therefore the study results reported use these second order constructs.

Figure A2-3. Formative Second Order Construct Assessment

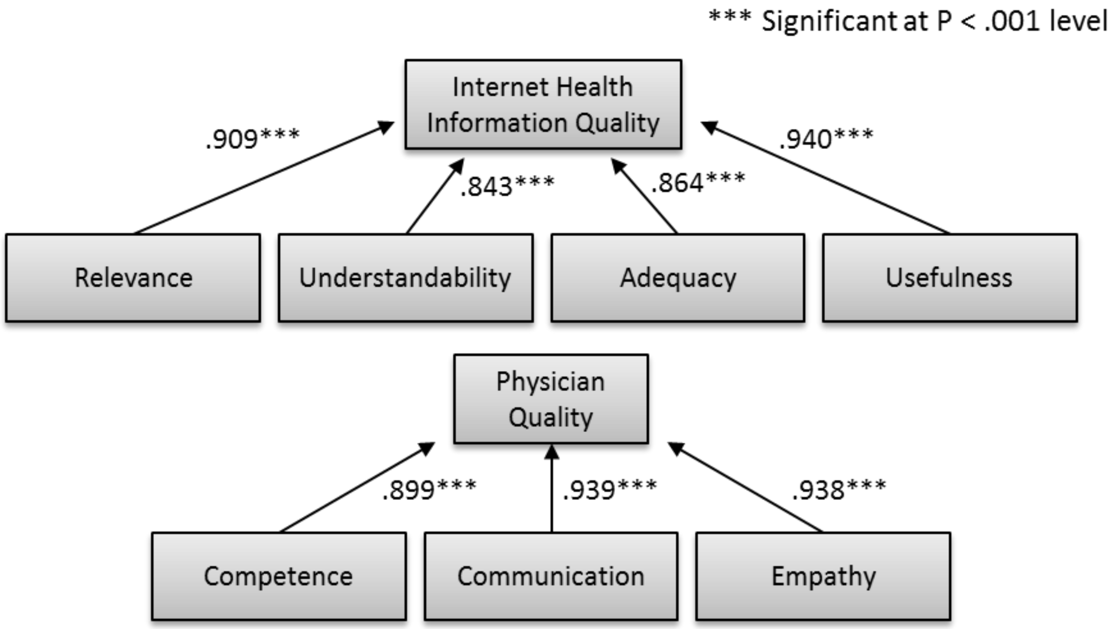

Statistical Analysis – Measurement Model

All first-order constructs in the research model were reflective constructs and therefore the methods to assess these constructs followed Gotz et al [iii], Gefen and Straub [iv], Fornell and Larcker [v] and Straub et al [vi]. Indicator convergent and discriminant validity were tested to ascertain whether any indicators warranted removal due to cross-loading issues. Gefen and Straub [iv] suggest the removal of any indicators that do not load by a factor of .1

higher on their theoretically assigned construct than on another construct in the model. The results demonstrate that no indicators needed to be removed, and the final set of indicators, all of which loaded on their own theoretically assigned constructs by a factor of .1 or greater are included in Table A2-1. The Compliance construct was not included in these results as it consisted of a calculated single measure and therefore results in a loading of 1.00. The Internet Health Information Quality and Physician Quality constructs were not included in these results as they are second order formative constructs. However, as an additional check, a full cross-loading analysis was completed that included all construct (i.e., both first and second order) indicators. No cross-loading issues were identified between the individual Compliance indicators and any other construct. In addition, no cross loading issues were identified between the first order indicators for Internet Health Information Quality and Physician Quality and any other construct. To assure content validity, this research made use of previously validated instruments that were carefully worded to address the context of this research study. Finally, indicator reliability was assessed by examining the indicator loadings and significance. All indicator loadings were above the .70 threshold [v] and were significant at the $p < .001$ level (see Table A2-1).

Table A2-1. Final reflective indicator loadings and cross loadings

	**PIA**	**PPC**	**Difference (largest – 2nd)**	**T-Statistic**	**Sig.**
**PIA1**	**.890**	.513	.377	38.669	p < .001
**PIA2**	**.960**	.597	.363	135.289	p < .001
**PIA3**	**.946**	.572	.374	93.692	p < .001
**PIA4**	**.960**	.594	.366	147.280	p < .001
**PPC1**	.570	**.844**	.274	21.025	p < .001
**PPC2**	.581	**.865**	.284	32.843	p < .001
**PPC3**	.475	**.860**	.385	33.612	p < .001
**PPC4**	.514	**.886**	.371	43.038	p < .001
**PPC5**	.480	**.873**	.393	37.704	p < .001

Note: Compliance is not included as it consisted of a composite of the individual indicator scores, Internet Health Information Quality and Physician Quality are not included as they are second order formative constructs; PIA=Perceived Information Asymmetry, PPC= Patient-Physician Concordance

To assess construct reliability, both Composite Reliability and Cronbachs α were examined. All Composite Reliability scores were well above the .80 threshold [v], with none of these scores lower than .937 (see Table A2-2). Similarly, all Cronbachs α scores were well above the .70 threshold [vii], and in fact were all above .90 with the lowest score of .916 (see Table A2-2). Finally construct validity was assessed by examining convergent and discriminant validity. Convergent validity was evaluated through examining Average Variance Extracted values to ensure they are all above the .50 threshold [v]. For all constructs, the Average Variance Extracted values exceeded this threshold, with the lowest Average Variance Extracted value of .749. To evaluate discriminant validity, a comparison of the square root of each construct's Average Variance Extracted versus the correlations that construct has

with other constructs in the model was completed (see Table A2-2). For all constructs, the square root of the Average Variance Extracted was larger than its correlations with other constructs in the model, providing evidence of discriminant validity [v]. Overall, the assessment of the measurement model provides strong evidence as to the reliability and validity of the indicators and constructs used.

Table A2-2. Construct Descriptives, Reliability, Validity, Correlations and AVEs

	Mean	SD	AVE	CR	Cronbachs α	COMPLY	PPC	PIA
**Compliance**	6.440	.794	1.000	n/a	n/a	**1.000**		
**PPC**	6.283	.867	.749	.937	.916	.561	**.866**	
**PIA**	6.148	.992	.883	.968	.956	.450	.607	**.940**

Note: Bolded diagonal elements represent square root of the AVE; SD=Standard Deviation, AVE=Average Variance Extracted, CR=Composite Reliability, PPC=Patient-Physician Concordance, PIA=Perceived Information Asymmetry

To ensure there were no multicollinearity issues with this research study, an examination of the bivariate correlations between the constructs in the model was completed. This examination showed no correlations larger than .607 (see Table A2-2), providing evidence that there are no multicollinearity issues [viii]. As an additional assessment of multicollinearity, an examination of the Tolerance and Variance Inflation Factor statistics was completed. For all constructs in the model, tolerance values were greater than the .01 threshold [viii] and all Variance Inflation Factors were below the commonly cited 3.3 threshold [ix] providing additional evidence that multicollinearity is not an issue.

Proactive methods were taken in an effort to ensure common method bias was not an issue for this research study. Survey items were ordered to control for priming effects by placing the endogenous construct items prior to the other construct items as per Podsakoff et al [x]. In addition, respondents were assured of the anonymity of their responses, as providing assurance of anonymity is a technique to control common method bias [x]. Once all data was gathered, the traditional and well-known Harman's One-Factor Test was applied. The results of this analysis indicated no presence of common method bias, with the Principal Components Analyses showing seven factors emerging (all with Eigen values greater than 1), with the first factor only accounting for 36.35% of the variance. While the Harman's One-Factor Test is not always definitive, it can be concluded that common method bias is not likely to be an issue for this research.

---

[i] *Pavlou PA, Gefen D. Psychological Contract Violation in Online Marketplaces: Antecedents, Consequences, and Moderating Role. Information Systems Research 2005 Dec; 16(4):372-399*

[ii] *Diamantopoulos A, Winklhofer HM. Index construction with formative indicators: an alternative to scale development. Journal of Marketing Research 2001 May; 38(2):269-277*

[iii] *Götz O, Liehr-Gobbers K, Krafft M. Evaluation of Structural Equation Models using the Partial Least Squares (PLS) approach. In: Esposito-Vinzi V, Chin WW, Henseler J, Wang H, editors. Handbook of Partial Least Squares. Berlin: Springer-Verlag; 2010. 691-711*

[iv] *Gefen D, Straub D. A practical guide to factorial validity using PLS-Graph: tutorial and annotated example. Communications of the Association for Information Systems 2005 Jul 21; 16(5):91-109*

[v] *Fornell C, Larcker DF. Evaluating Structural Equation Models with Unobservable Variables and Measurement Error. Journal of Marketing Research 1981 Feb; 18(1):39*

[vi] *Straub D, Boudreau MC, Gefen D. Validation guidelines for IS positivist research. Communications of the Association for Information Systems 2004 Mar 10; 13(24):380-427*

[vii] *Cronbach LJ. Coefficient alpha and the internal structure of tests. Psychometrika 1951 Sept; 16(3):297-334*

[viii] *Meyers LS, Gamst G, Guarino AJ. Applied Multivariate Research: Design and Interpretation. Thousand Oaks, CA: Sage Publications, Inc; 2006.*

[ix] *Petter S, Straub D, Rai A. Specifying formative constructs in Information Systems research. MIS Quarterly 2007 Dec; 31(4):623-656*

[x] *Podsakoff Philip M, MacKenzie Scott B, Lee Jeong-Yeon, Podsakoff Nathan P. Common method biases in behavioral research: a critical review of the literature and recommended remedies. J Appl Psychol 2003 Oct; 88(5):879-903*
